# Supplementary figures and images for: BDNF/TrkB signaling endosomes in axons coordinate CREB/mTOR activation and protein synthesis in the cell body to induce dendritic growth in cortical neurons
Source: eLife. 2023 Feb 24;12:e77455. doi: 10.7554/eLife.77455 (PMC9977295; doi:10.7554/eLife.77455)

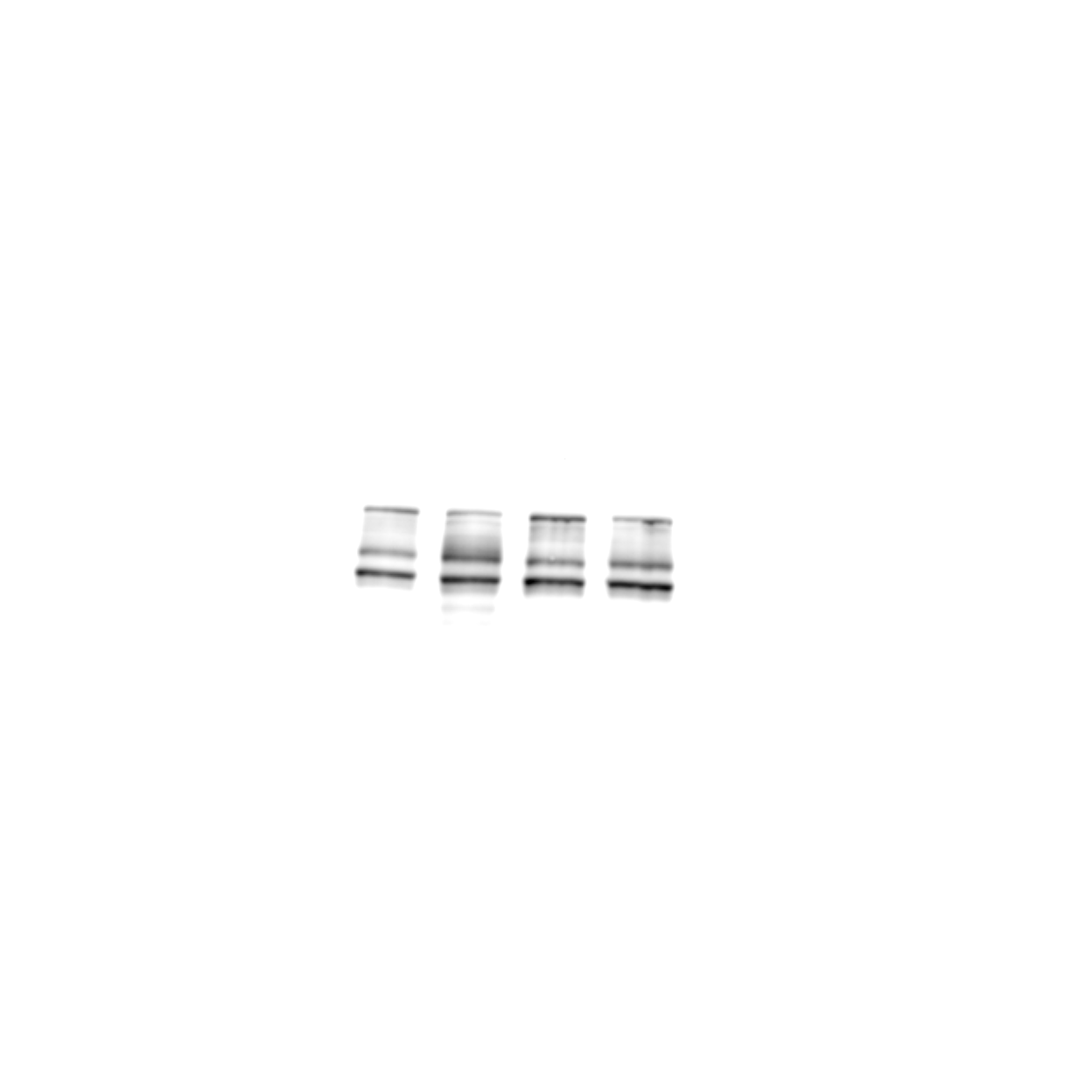

Supplement: Figure 5—figure supplement 1—source data 1. — Original Western blots supporting Figure 5—figure supplement 1. This source data contains the original Western blots that supports the Figure 5—figure supplement 1. The images are separated in folders each one corresponding to independent experiments (N1, N2, N3). In the original pictures, the order of lines is the same than in Figure 5—figure supplement 1B and the name of the file indicates the antibody used to develop the nitrocellulose membrane. [file elife-77455-fig5-figsupp1-data1.zip › Supplementary Figure 3 WB/N1/ptrkb816 20190104_120116_Ch.tif]

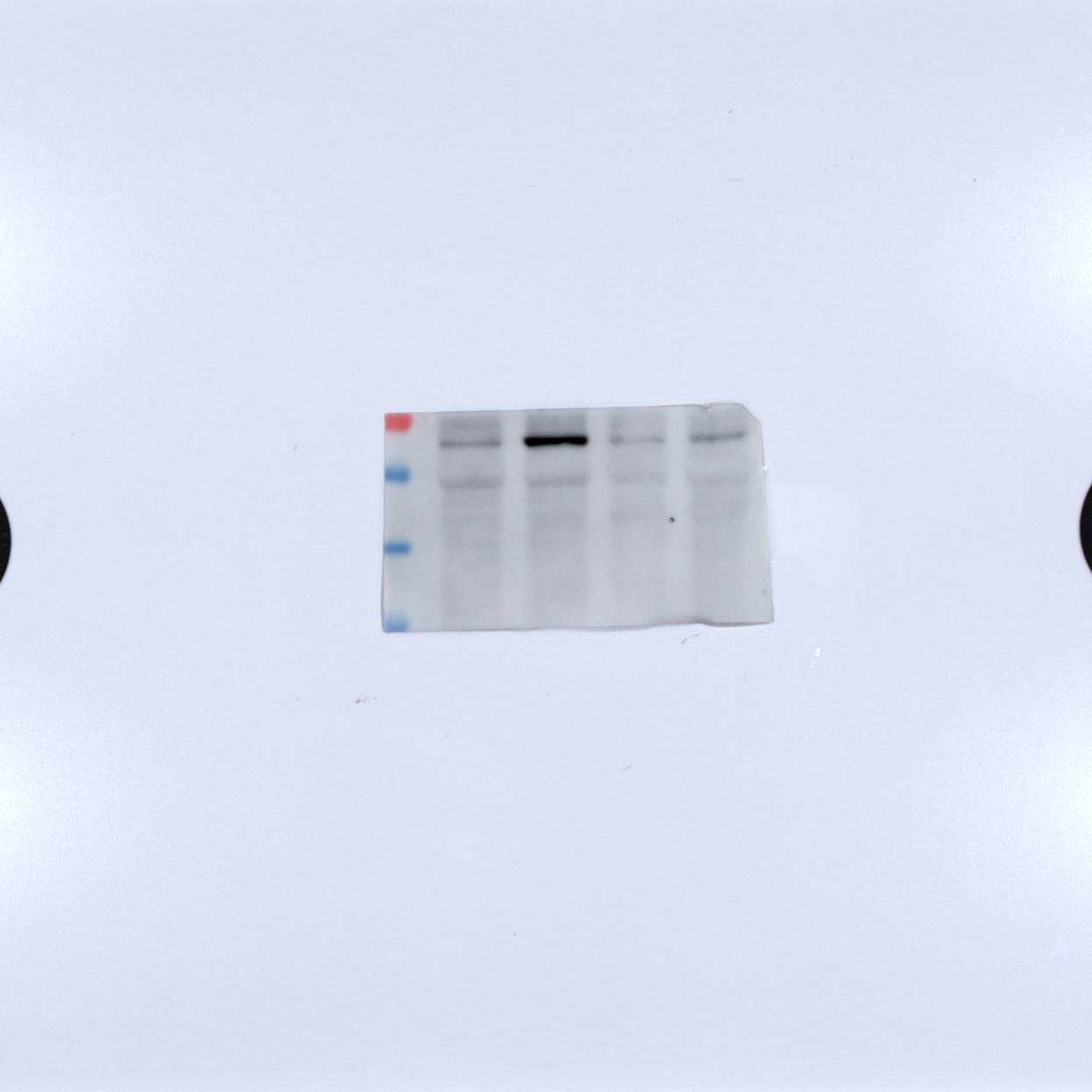

Supplement: Figure 5—figure supplement 1—source data 1. — Original Western blots supporting Figure 5—figure supplement 1. This source data contains the original Western blots that supports the Figure 5—figure supplement 1. The images are separated in folders each one corresponding to independent experiments (N1, N2, N3). In the original pictures, the order of lines is the same than in Figure 5—figure supplement 1B and the name of the file indicates the antibody used to develop the nitrocellulose membrane. [file elife-77455-fig5-figsupp1-data1.zip › Supplementary Figure 3 WB/N1/pakt 20181228_120934_Ch+Marker.tif]

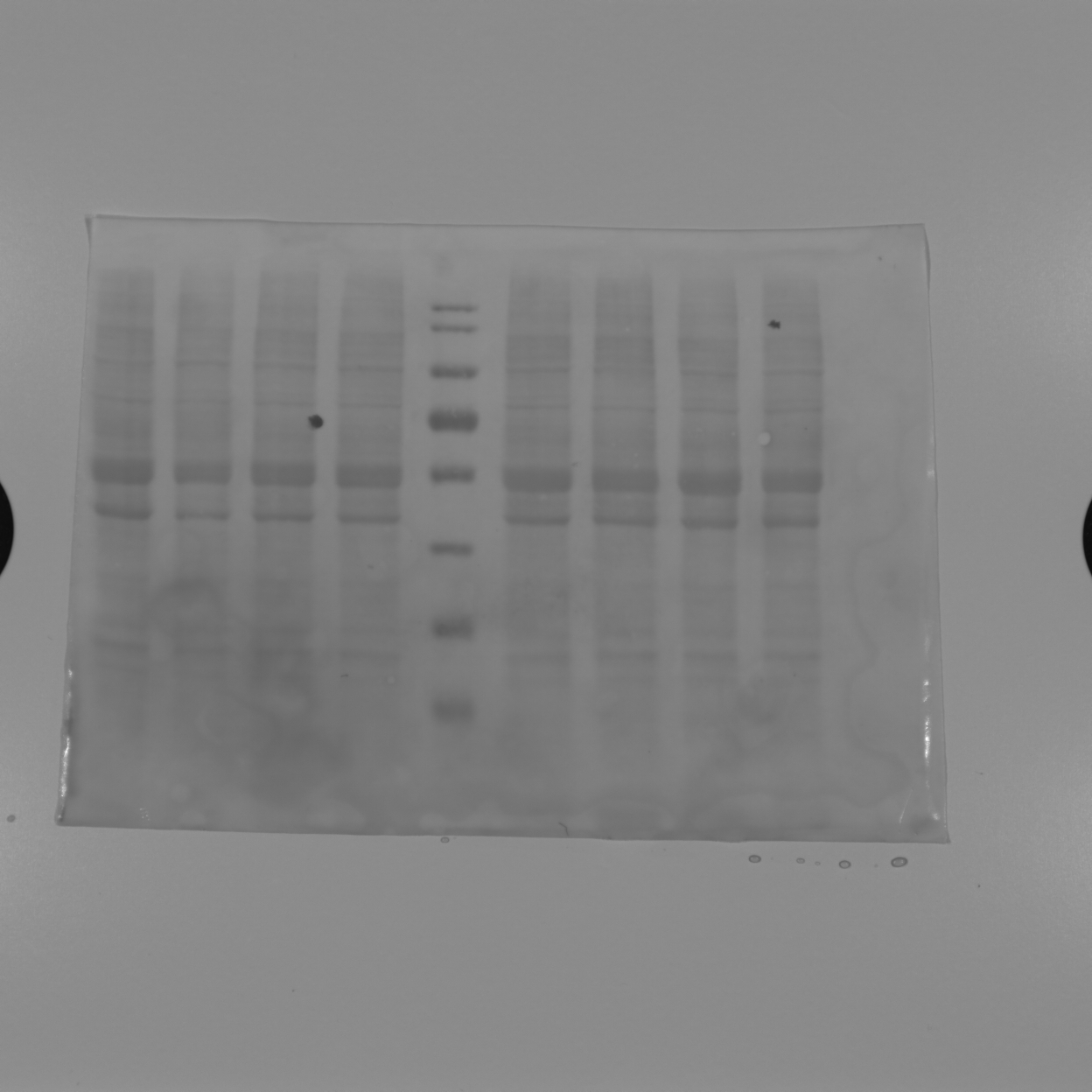

Supplement: Figure 5—figure supplement 1—source data 1. — Original Western blots supporting Figure 5—figure supplement 1. This source data contains the original Western blots that supports the Figure 5—figure supplement 1. The images are separated in folders each one corresponding to independent experiments (N1, N2, N3). In the original pictures, the order of lines is the same than in Figure 5—figure supplement 1B and the name of the file indicates the antibody used to develop the nitrocellulose membrane. [file elife-77455-fig5-figsupp1-data1.zip › Supplementary Figure 3 WB/N1/ponceau_red 20181227_150017_Co.tif]

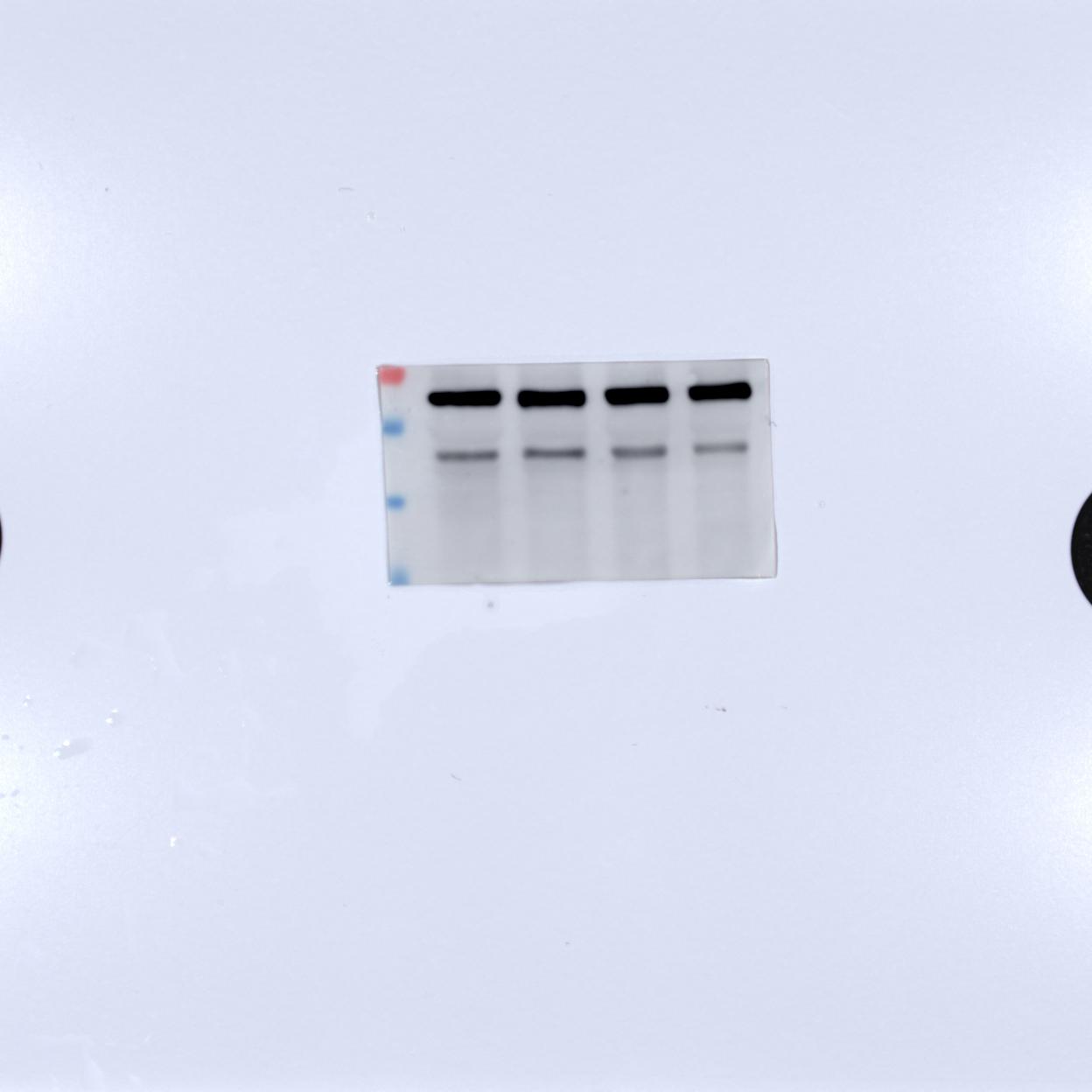

Supplement: Figure 5—figure supplement 1—source data 1. — Original Western blots supporting Figure 5—figure supplement 1. This source data contains the original Western blots that supports the Figure 5—figure supplement 1. The images are separated in folders each one corresponding to independent experiments (N1, N2, N3). In the original pictures, the order of lines is the same than in Figure 5—figure supplement 1B and the name of the file indicates the antibody used to develop the nitrocellulose membrane. [file elife-77455-fig5-figsupp1-data1.zip › Supplementary Figure 3 WB/N1/akt_total 20181228_121531_Ch+Marker.jpg]

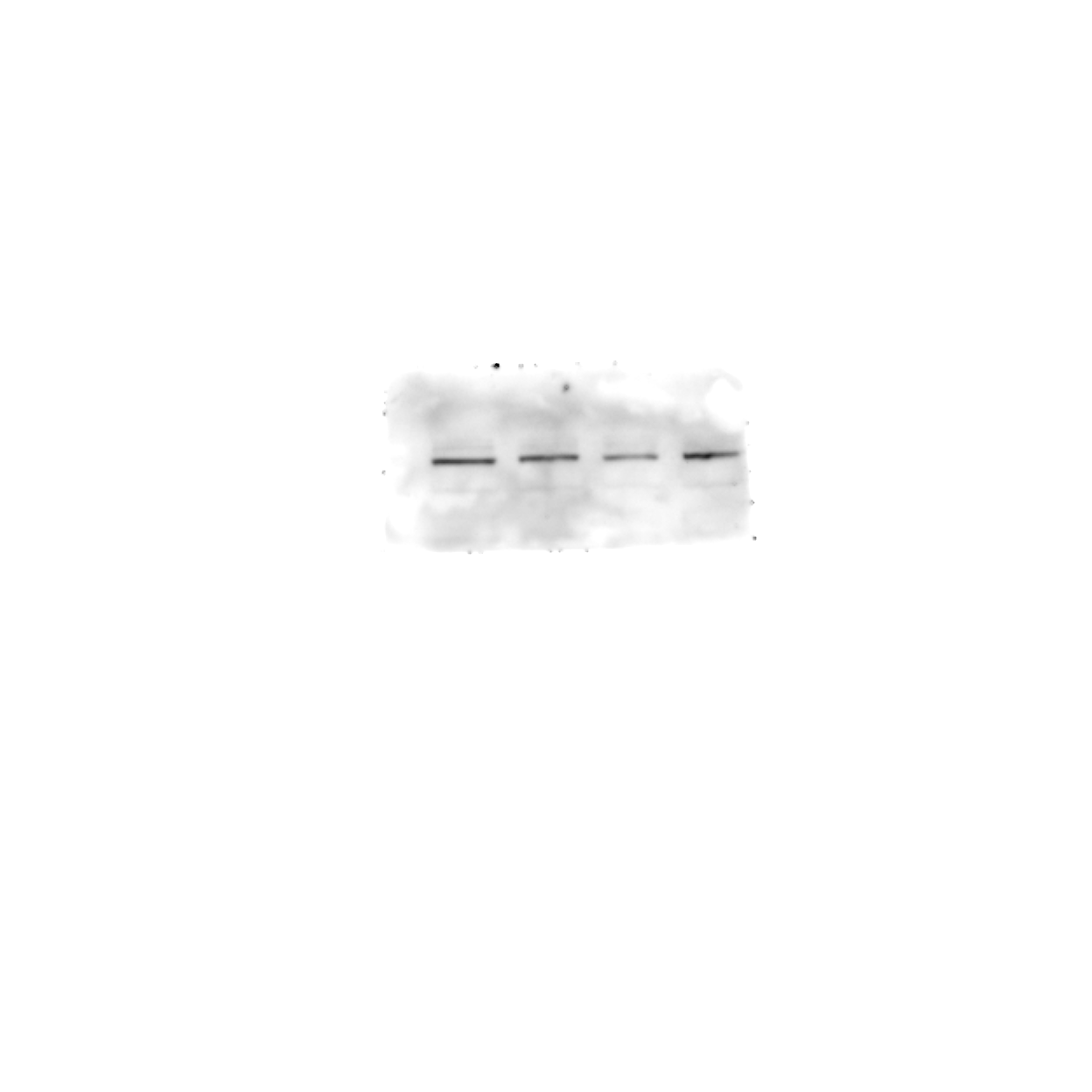

Supplement: Figure 5—figure supplement 1—source data 1. — Original Western blots supporting Figure 5—figure supplement 1. This source data contains the original Western blots that supports the Figure 5—figure supplement 1. The images are separated in folders each one corresponding to independent experiments (N1, N2, N3). In the original pictures, the order of lines is the same than in Figure 5—figure supplement 1B and the name of the file indicates the antibody used to develop the nitrocellulose membrane. [file elife-77455-fig5-figsupp1-data1.zip › Supplementary Figure 3 WB/N1/trkb total 20190104_124721_Ch.tif]

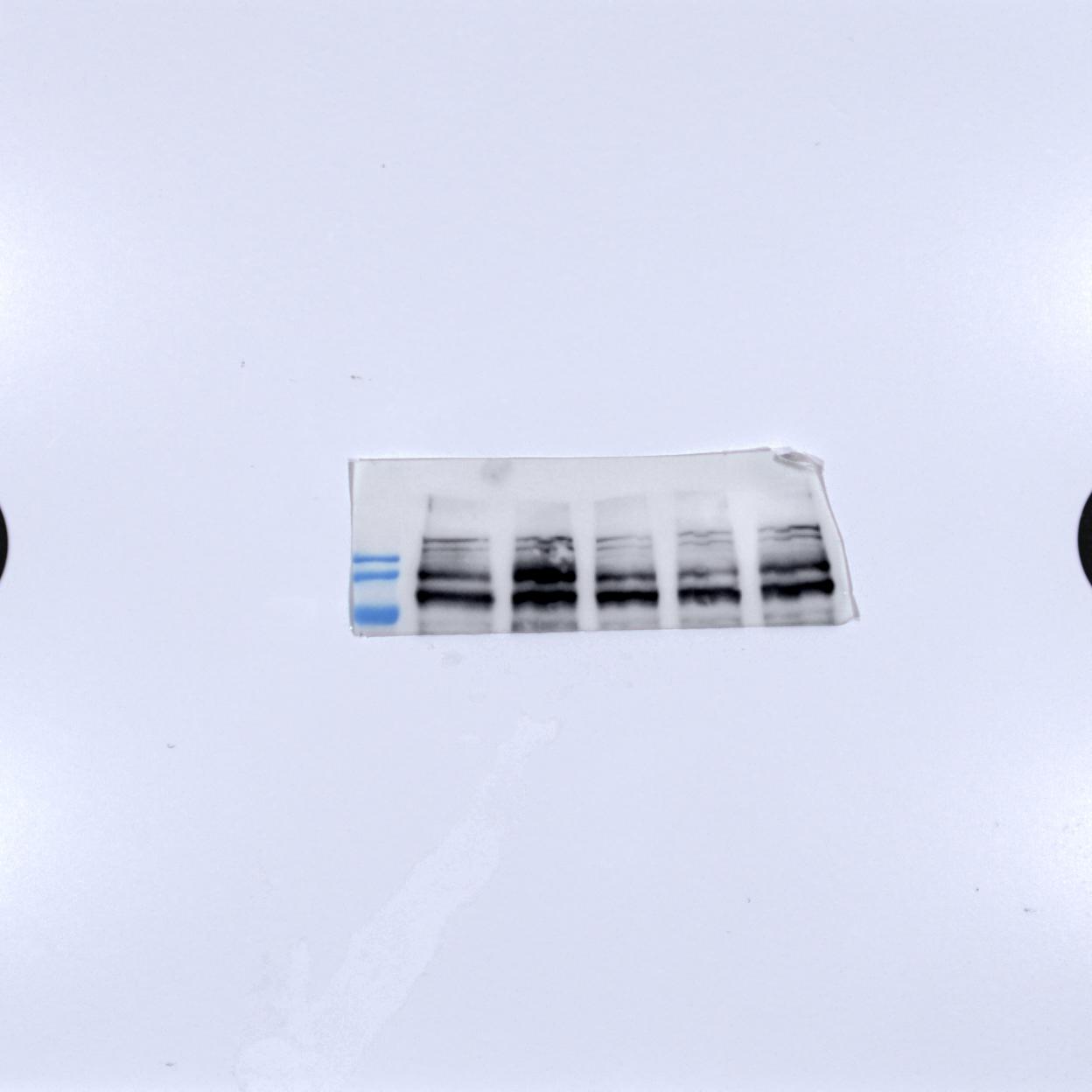

Supplement: Figure 5—figure supplement 1—source data 1. — Original Western blots supporting Figure 5—figure supplement 1. This source data contains the original Western blots that supports the Figure 5—figure supplement 1. The images are separated in folders each one corresponding to independent experiments (N1, N2, N3). In the original pictures, the order of lines is the same than in Figure 5—figure supplement 1B and the name of the file indicates the antibody used to develop the nitrocellulose membrane. [file elife-77455-fig5-figsupp1-data1.zip › Supplementary Figure 3 WB/N3/ptrkB 20190621_143341_Ch+Marker.jpg]

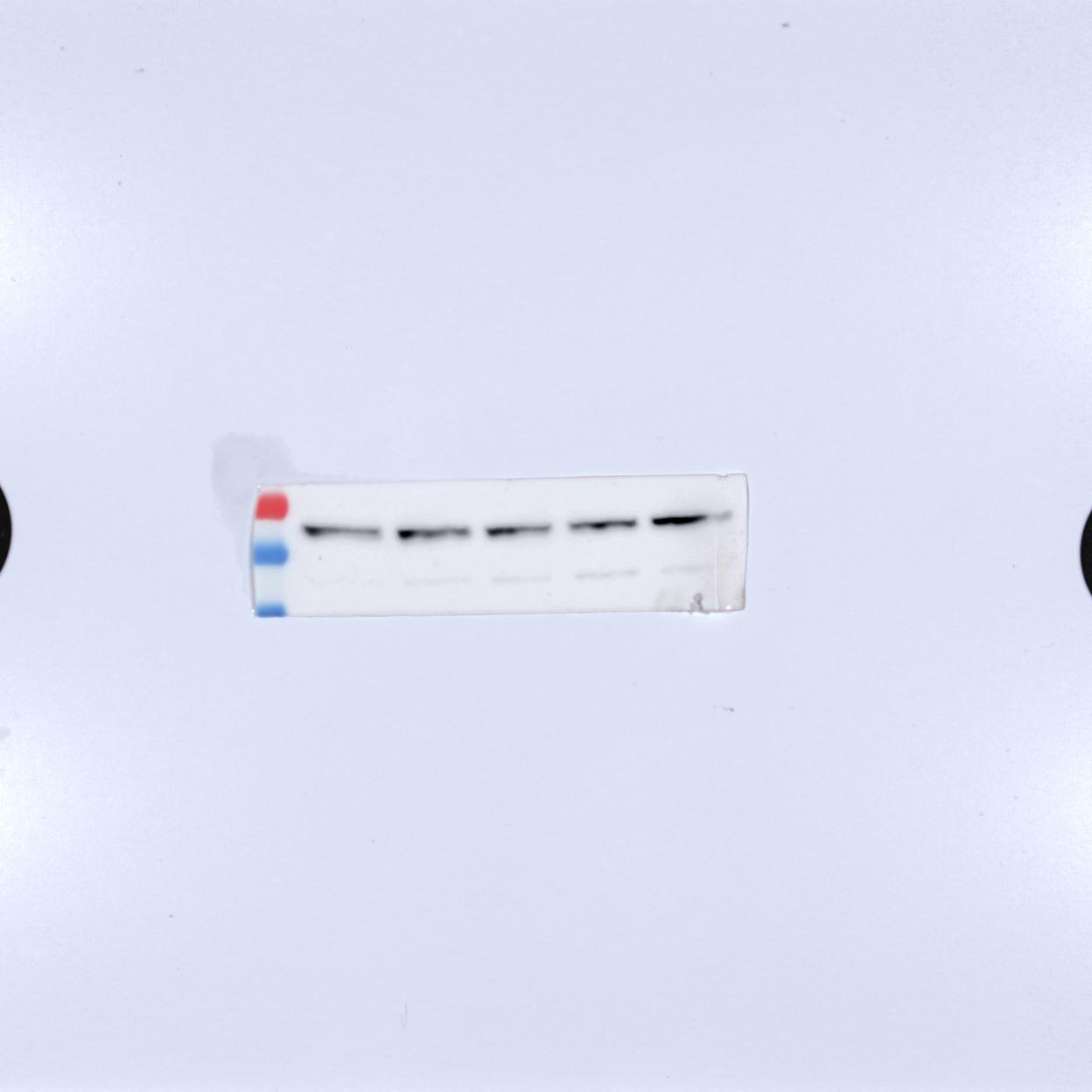

Supplement: Figure 5—figure supplement 1—source data 1. — Original Western blots supporting Figure 5—figure supplement 1. This source data contains the original Western blots that supports the Figure 5—figure supplement 1. The images are separated in folders each one corresponding to independent experiments (N1, N2, N3). In the original pictures, the order of lines is the same than in Figure 5—figure supplement 1B and the name of the file indicates the antibody used to develop the nitrocellulose membrane. [file elife-77455-fig5-figsupp1-data1.zip › Supplementary Figure 3 WB/N3/akt total ln3 20190705_134305_Ch+Marker.jpg]

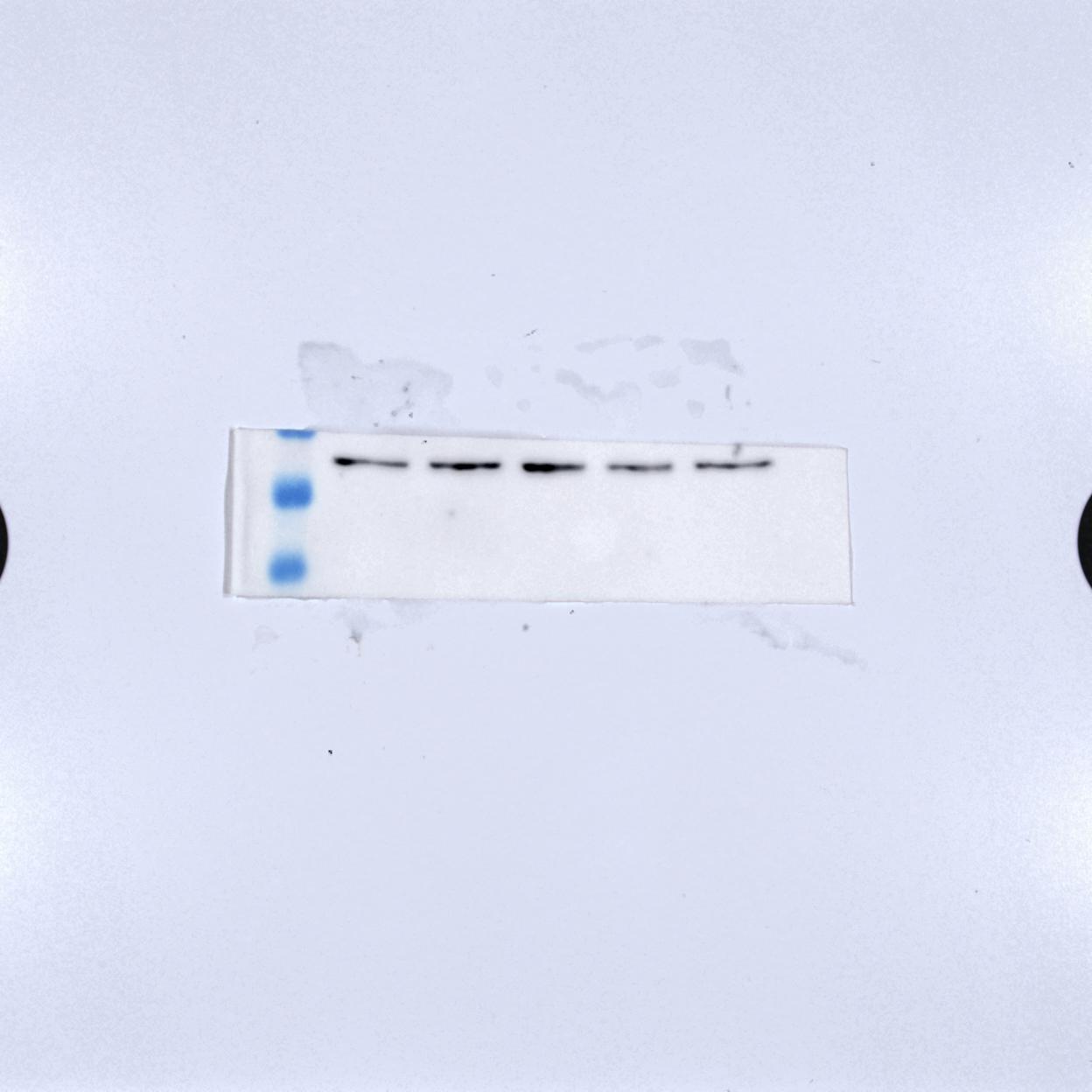

Supplement: Figure 5—figure supplement 1—source data 1. — Original Western blots supporting Figure 5—figure supplement 1. This source data contains the original Western blots that supports the Figure 5—figure supplement 1. The images are separated in folders each one corresponding to independent experiments (N1, N2, N3). In the original pictures, the order of lines is the same than in Figure 5—figure supplement 1B and the name of the file indicates the antibody used to develop the nitrocellulose membrane. [file elife-77455-fig5-figsupp1-data1.zip › Supplementary Figure 3 WB/N3/gapkoin3 20190621_135640_Ch+Marker.jpg]

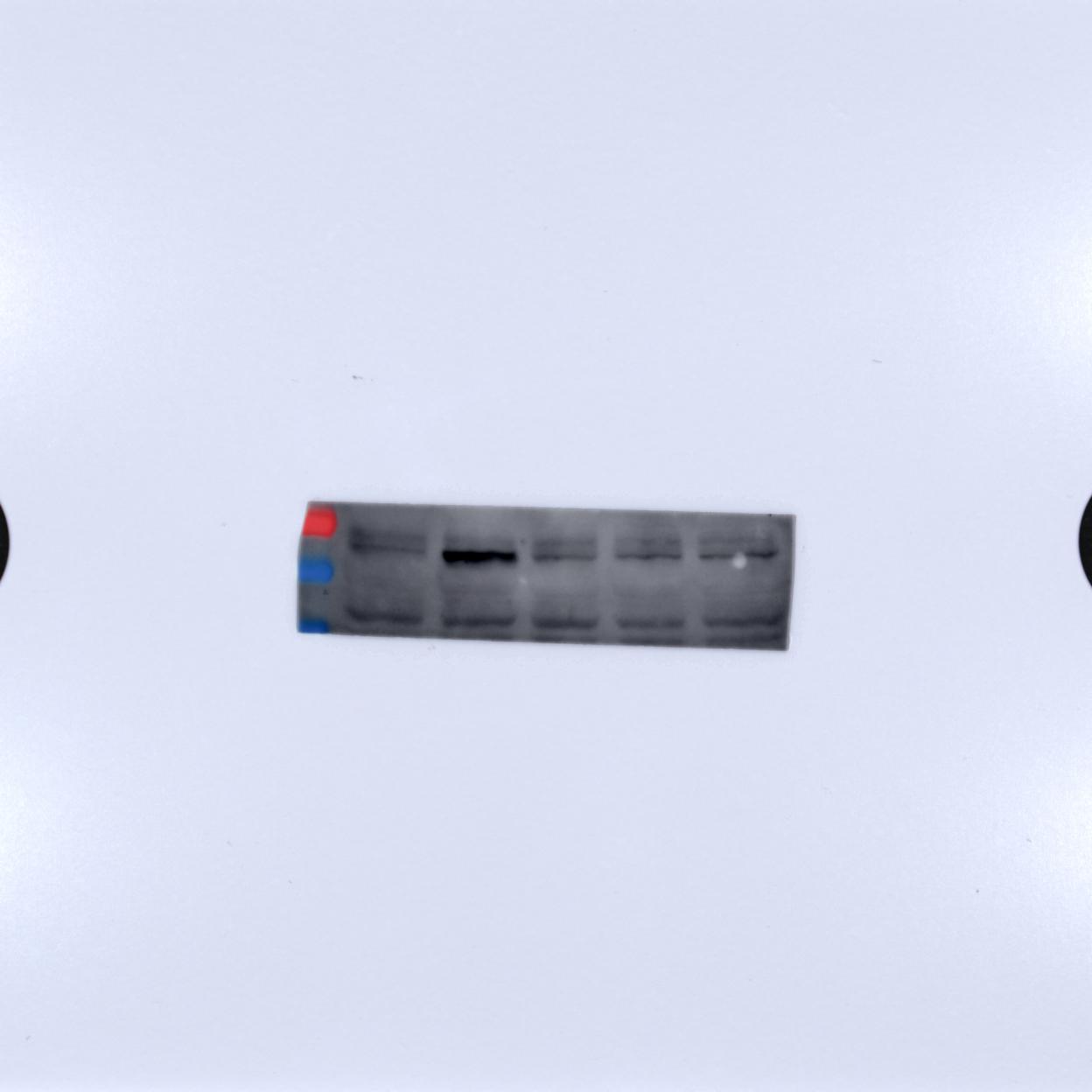

Supplement: Figure 5—figure supplement 1—source data 1. — Original Western blots supporting Figure 5—figure supplement 1. This source data contains the original Western blots that supports the Figure 5—figure supplement 1. The images are separated in folders each one corresponding to independent experiments (N1, N2, N3). In the original pictures, the order of lines is the same than in Figure 5—figure supplement 1B and the name of the file indicates the antibody used to develop the nitrocellulose membrane. [file elife-77455-fig5-figsupp1-data1.zip › Supplementary Figure 3 WB/N3/pakt 20190621_134731_Ch+Marker.jpg]

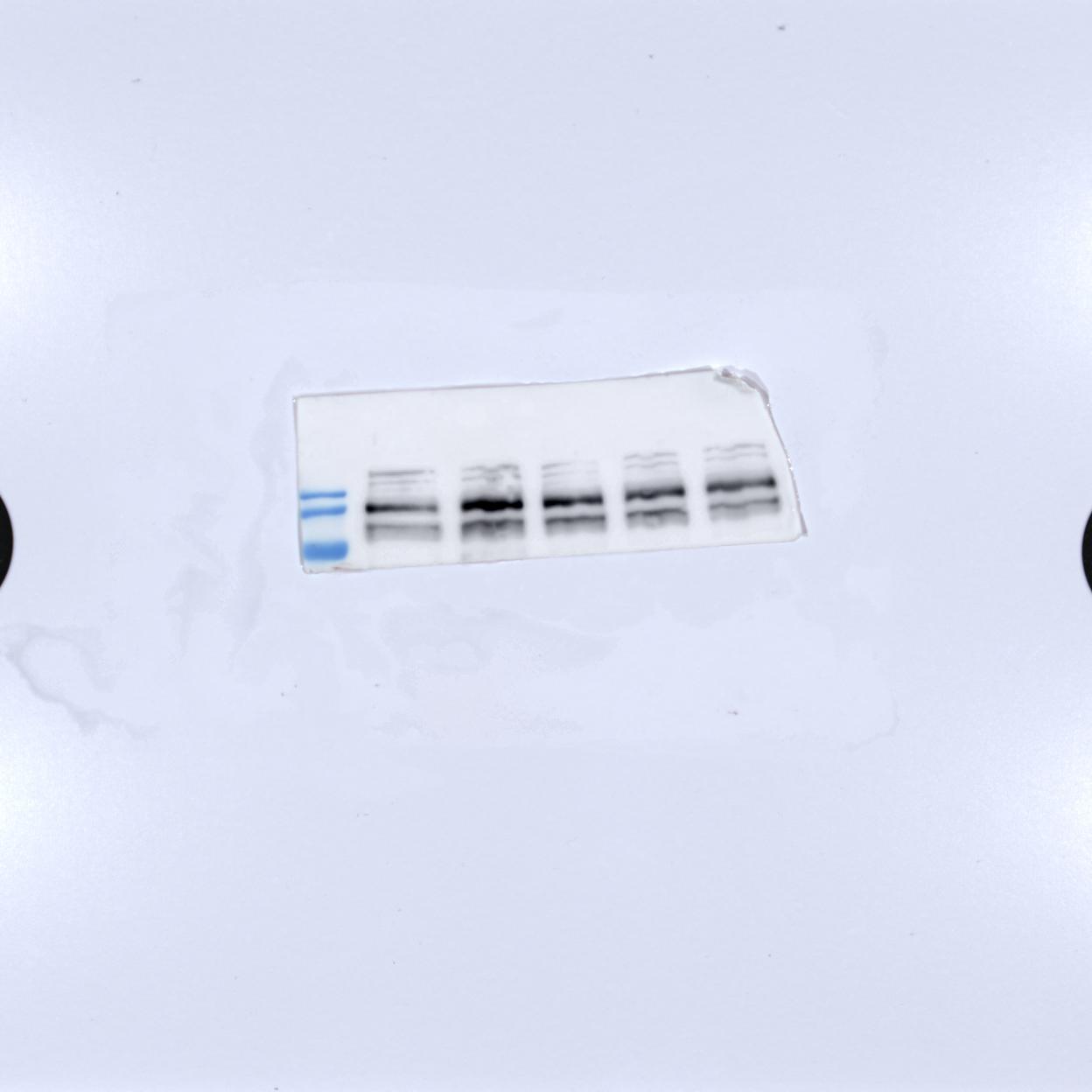

Supplement: Figure 5—figure supplement 1—source data 1. — Original Western blots supporting Figure 5—figure supplement 1. This source data contains the original Western blots that supports the Figure 5—figure supplement 1. The images are separated in folders each one corresponding to independent experiments (N1, N2, N3). In the original pictures, the order of lines is the same than in Figure 5—figure supplement 1B and the name of the file indicates the antibody used to develop the nitrocellulose membrane. [file elife-77455-fig5-figsupp1-data1.zip › Supplementary Figure 3 WB/N3/trkB total n3 20190705_133722_Ch+Marker.jpg]

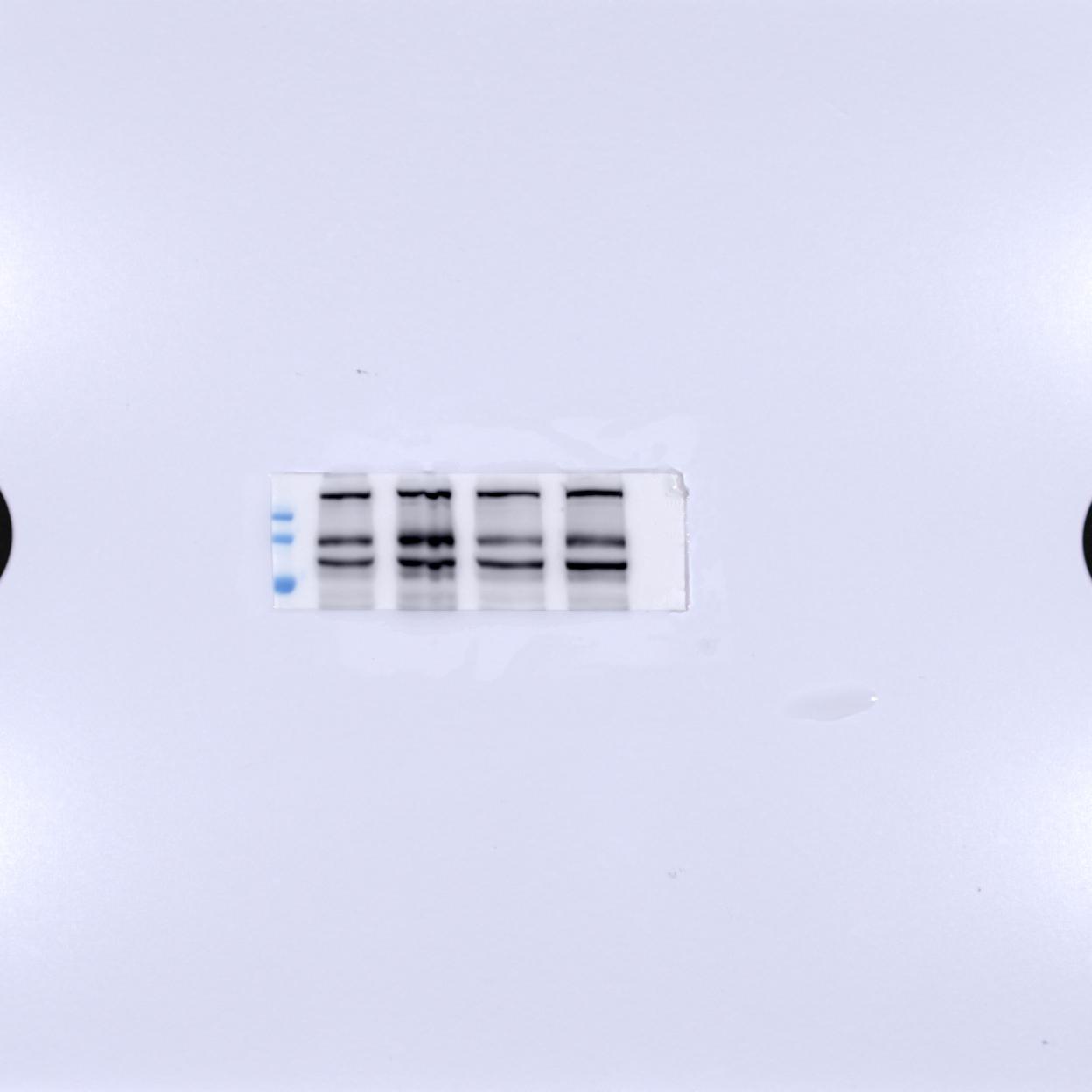

Supplement: Figure 5—figure supplement 1—source data 1. — Original Western blots supporting Figure 5—figure supplement 1. This source data contains the original Western blots that supports the Figure 5—figure supplement 1. The images are separated in folders each one corresponding to independent experiments (N1, N2, N3). In the original pictures, the order of lines is the same than in Figure 5—figure supplement 1B and the name of the file indicates the antibody used to develop the nitrocellulose membrane. [file elife-77455-fig5-figsupp1-data1.zip › Supplementary Figure 3 WB/N2/ptrkB n2 20190130_162713_Ch+Marker.jpg]

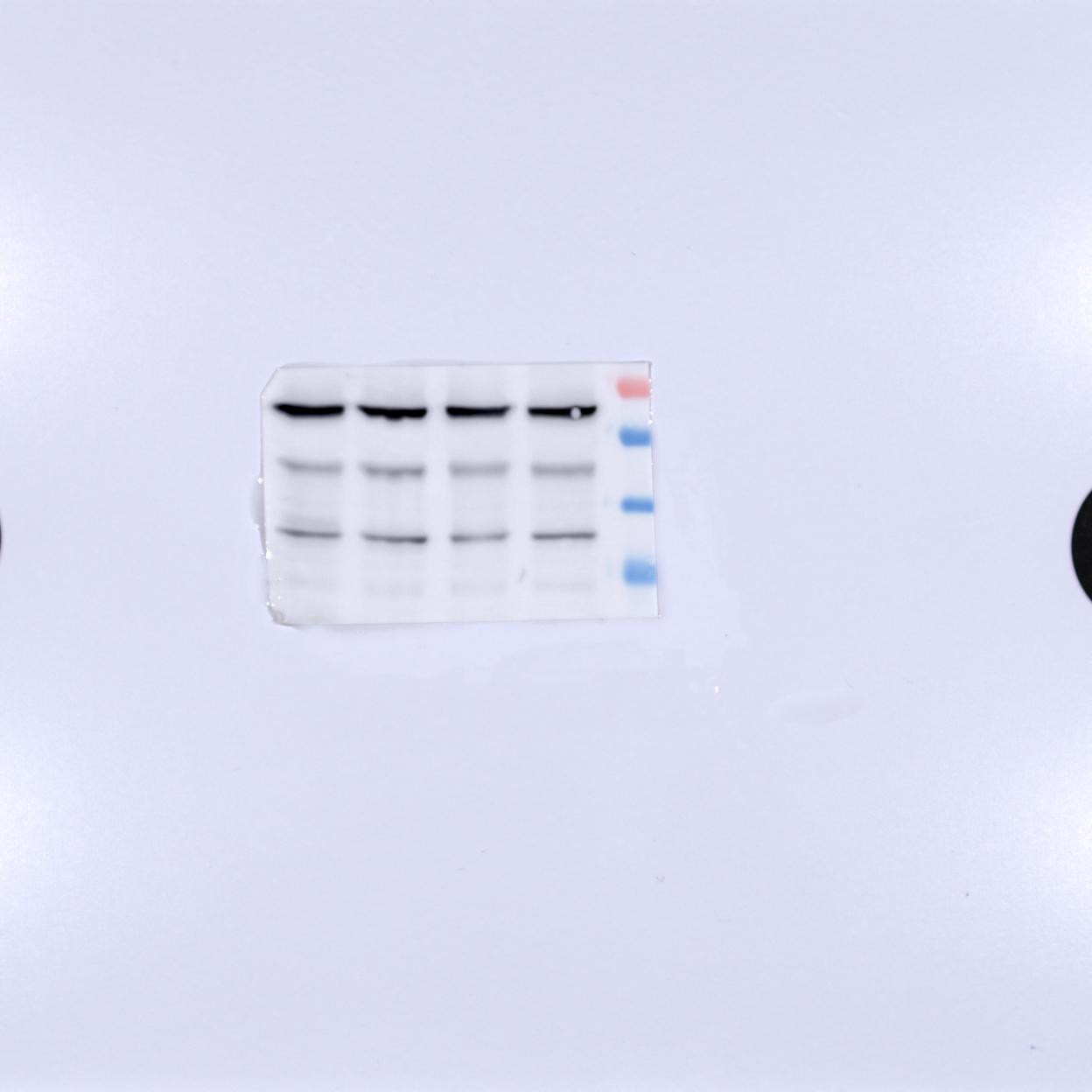

Supplement: Figure 5—figure supplement 1—source data 1. — Original Western blots supporting Figure 5—figure supplement 1. This source data contains the original Western blots that supports the Figure 5—figure supplement 1. The images are separated in folders each one corresponding to independent experiments (N1, N2, N3). In the original pictures, the order of lines is the same than in Figure 5—figure supplement 1B and the name of the file indicates the antibody used to develop the nitrocellulose membrane. [file elife-77455-fig5-figsupp1-data1.zip › Supplementary Figure 3 WB/N2/akt total n2 20190130_163647_Ch+Marker.jpg]

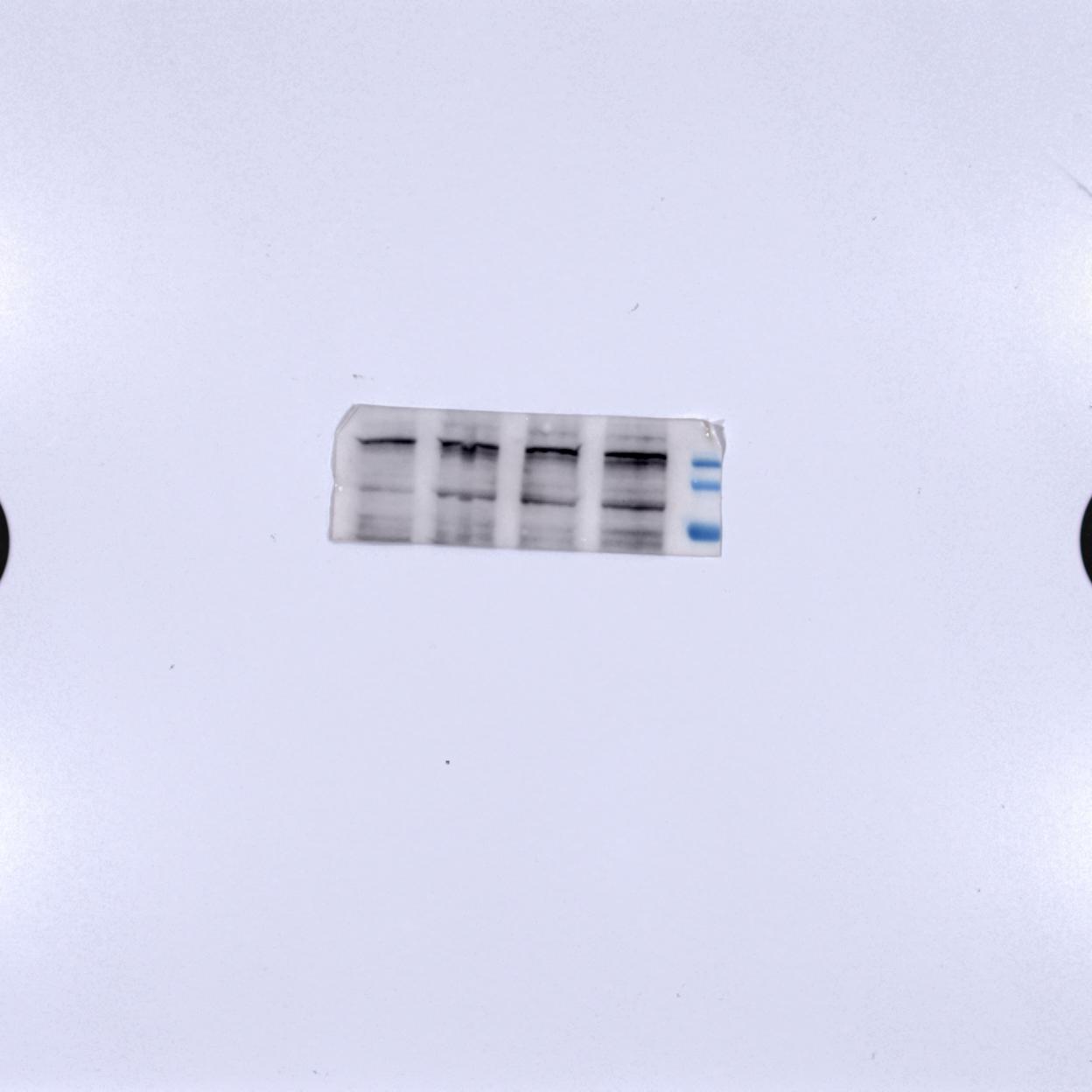

Supplement: Figure 5—figure supplement 1—source data 1. — Original Western blots supporting Figure 5—figure supplement 1. This source data contains the original Western blots that supports the Figure 5—figure supplement 1. The images are separated in folders each one corresponding to independent experiments (N1, N2, N3). In the original pictures, the order of lines is the same than in Figure 5—figure supplement 1B and the name of the file indicates the antibody used to develop the nitrocellulose membrane. [file elife-77455-fig5-figsupp1-data1.zip › Supplementary Figure 3 WB/N2/trkb total n2 20190130_165717_Ch+Marker.jpg]

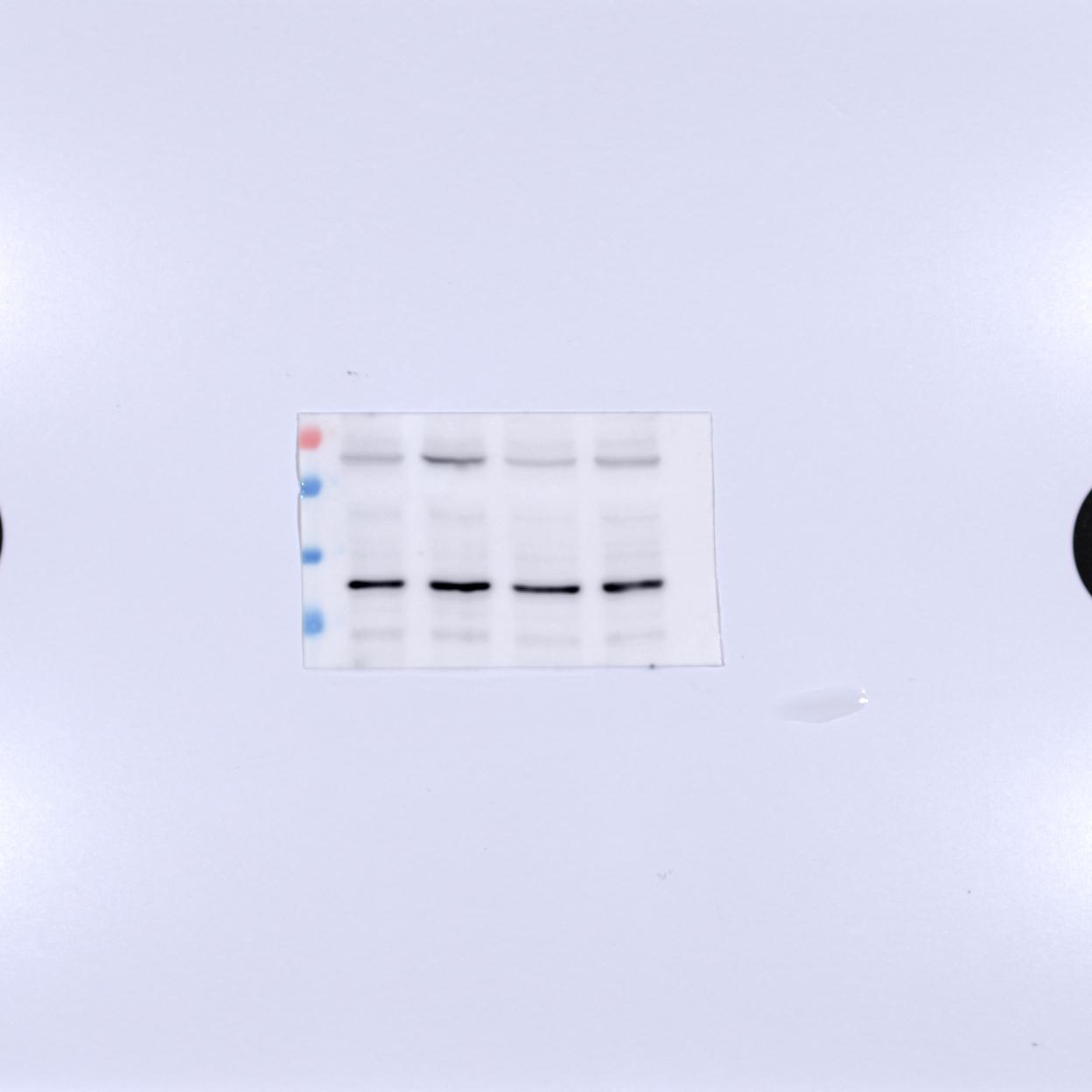

Supplement: Figure 5—figure supplement 1—source data 1. — Original Western blots supporting Figure 5—figure supplement 1. This source data contains the original Western blots that supports the Figure 5—figure supplement 1. The images are separated in folders each one corresponding to independent experiments (N1, N2, N3). In the original pictures, the order of lines is the same than in Figure 5—figure supplement 1B and the name of the file indicates the antibody used to develop the nitrocellulose membrane. [file elife-77455-fig5-figsupp1-data1.zip › Supplementary Figure 3 WB/N2/pakt n2 20190130_162057_Ch+Marker.jpg]

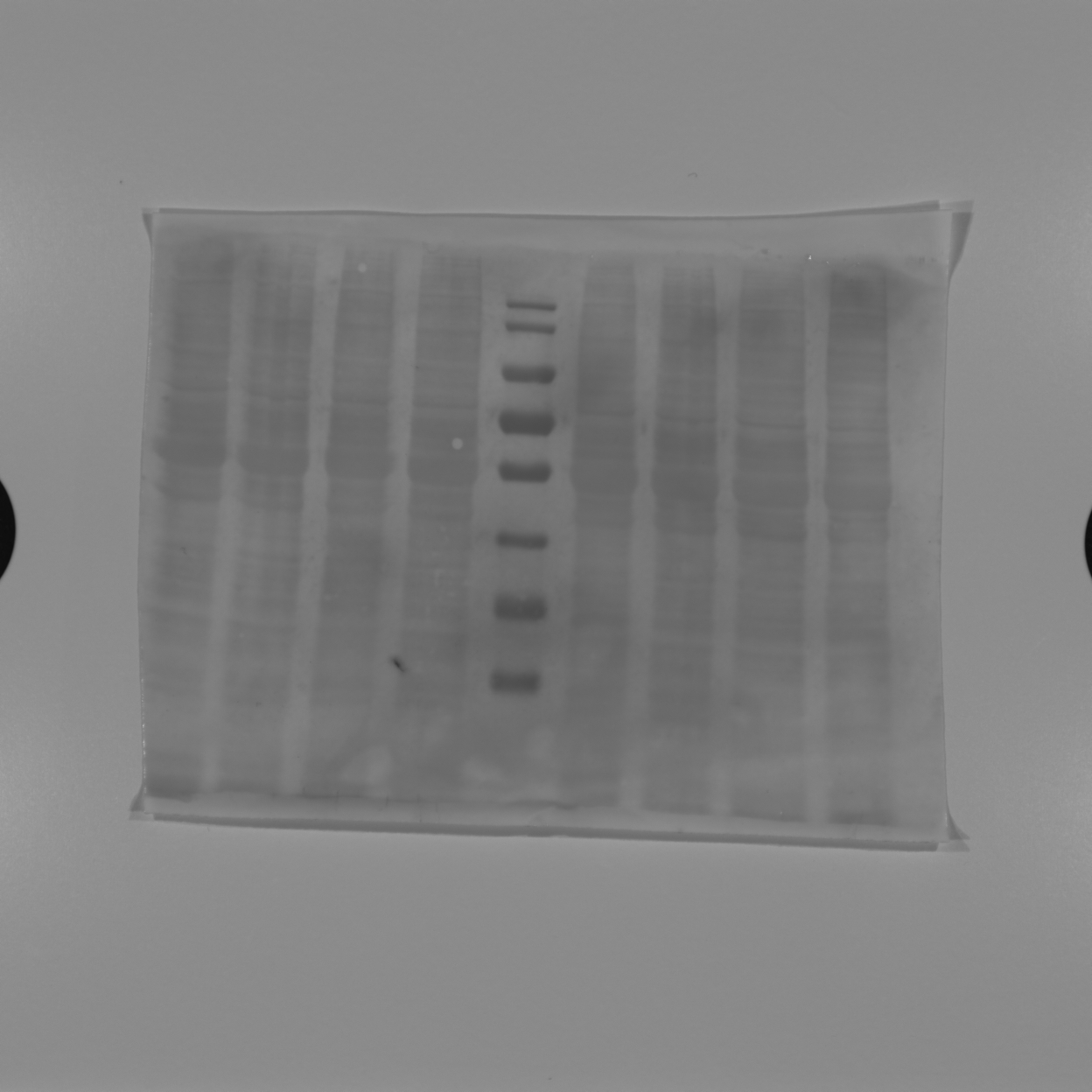

Supplement: Figure 5—figure supplement 1—source data 1. — Original Western blots supporting Figure 5—figure supplement 1. This source data contains the original Western blots that supports the Figure 5—figure supplement 1. The images are separated in folders each one corresponding to independent experiments (N1, N2, N3). In the original pictures, the order of lines is the same than in Figure 5—figure supplement 1B and the name of the file indicates the antibody used to develop the nitrocellulose membrane. [file elife-77455-fig5-figsupp1-data1.zip › Supplementary Figure 3 WB/N2/ponceau n2 nmp 20190129_150449_Co.tif]

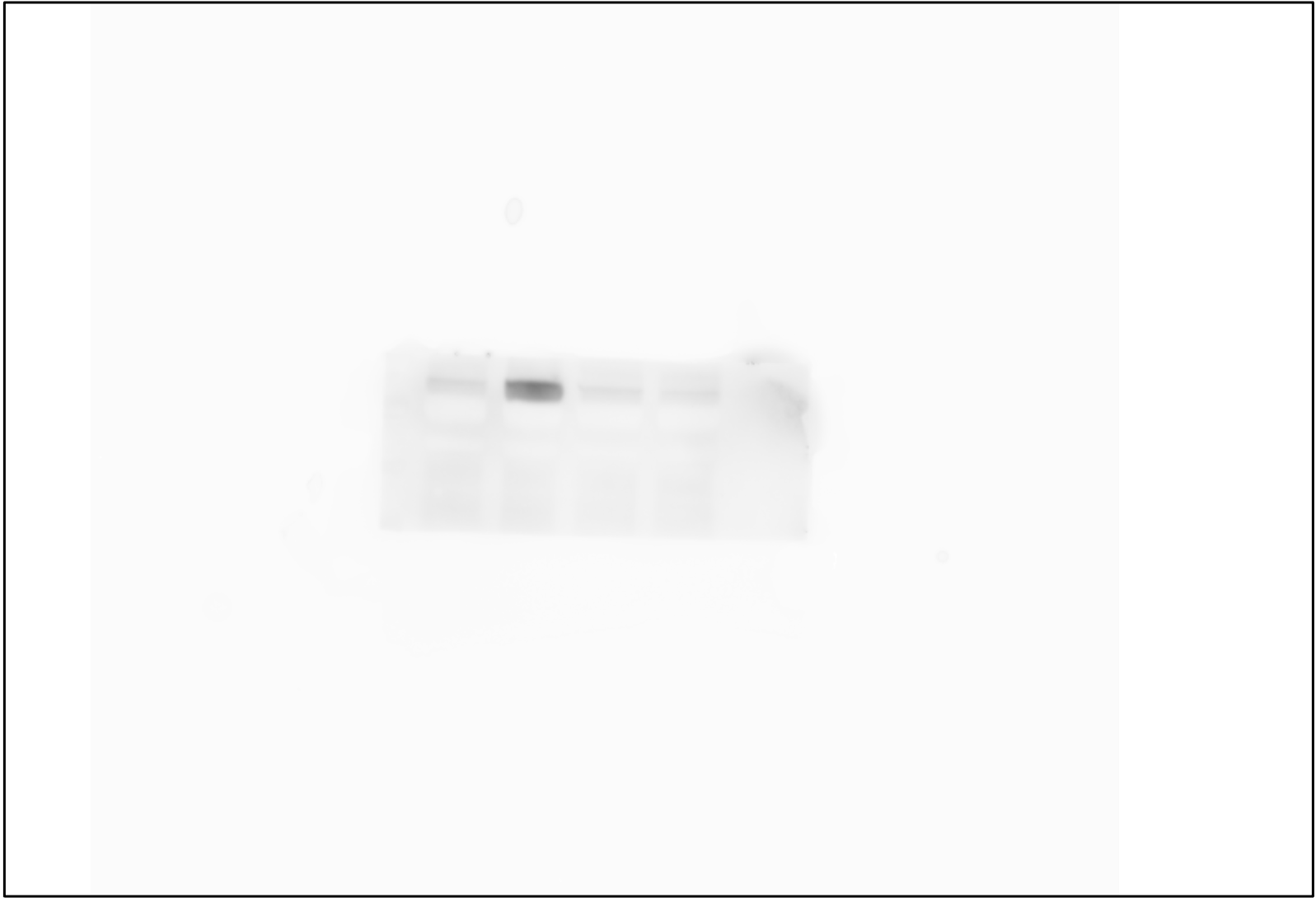

Supplement: Figure 7—figure supplement 2—source data 1. — In the original pictures, the order of lines is the same than in Figure 7—figure supplement 2 and the name of the file indicates the antibody used to develop the nitrocellulose membrane. [file elife-77455-fig7-figsupp2-data1.zip › Supplementary Figure 4 WB/pAkt_s473.png]

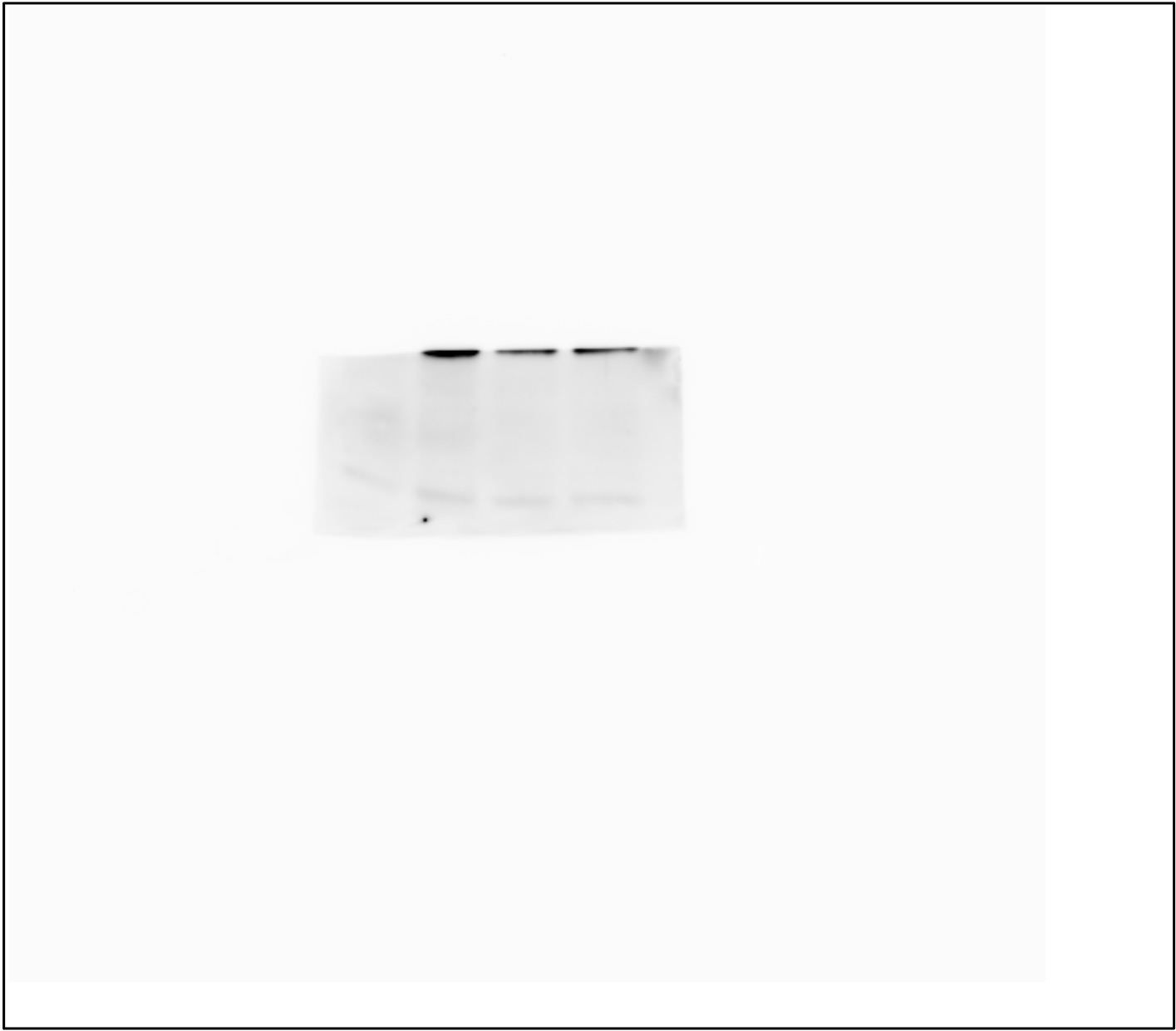

Supplement: Figure 7—figure supplement 2—source data 1. — In the original pictures, the order of lines is the same than in Figure 7—figure supplement 2 and the name of the file indicates the antibody used to develop the nitrocellulose membrane. [file elife-77455-fig7-figsupp2-data1.zip › Supplementary Figure 4 WB/pS6 s235_236.jpg]

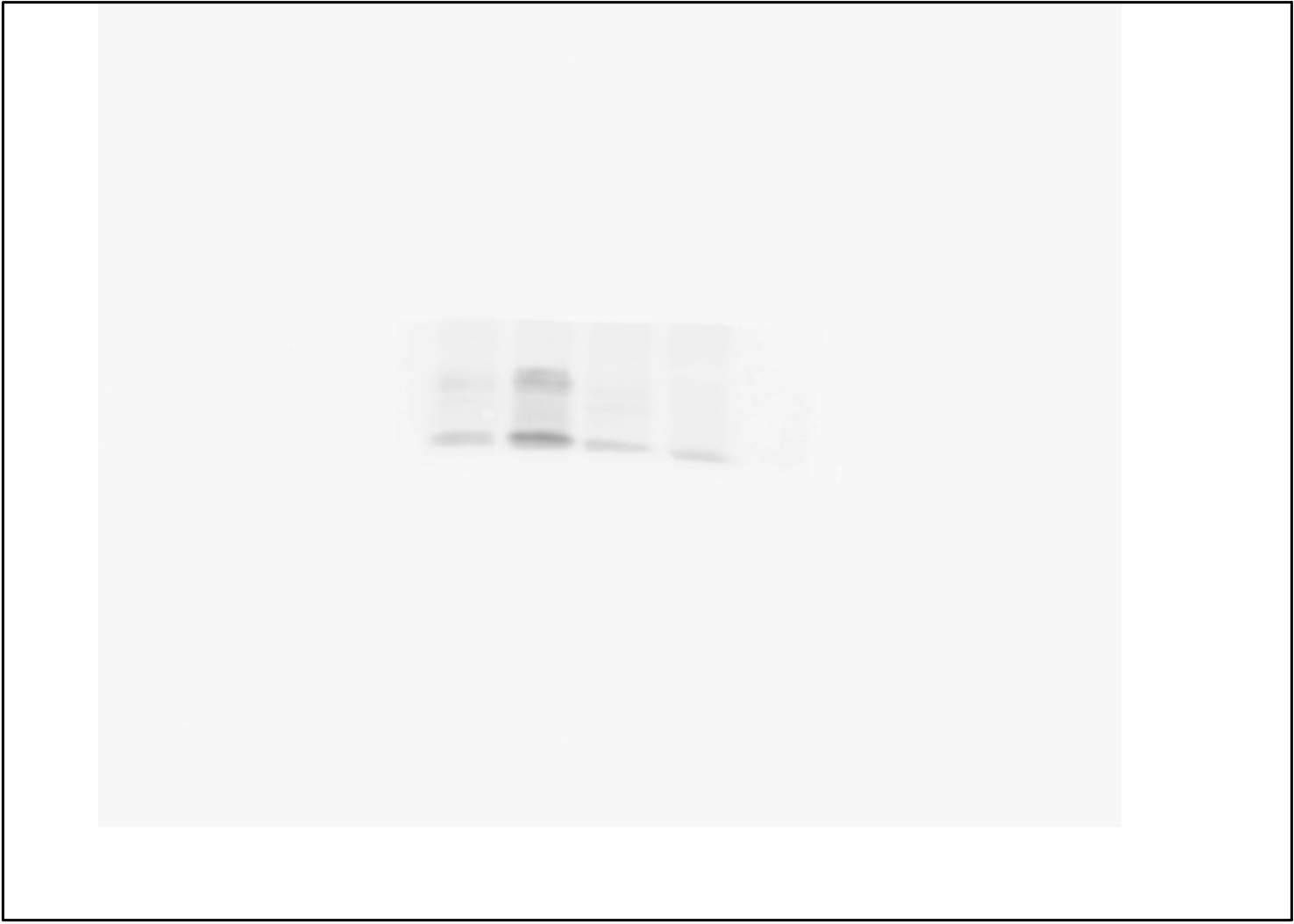

Supplement: Figure 7—figure supplement 2—source data 1. — In the original pictures, the order of lines is the same than in Figure 7—figure supplement 2 and the name of the file indicates the antibody used to develop the nitrocellulose membrane. [file elife-77455-fig7-figsupp2-data1.zip › Supplementary Figure 4 WB/p4EB-P1.jpg]

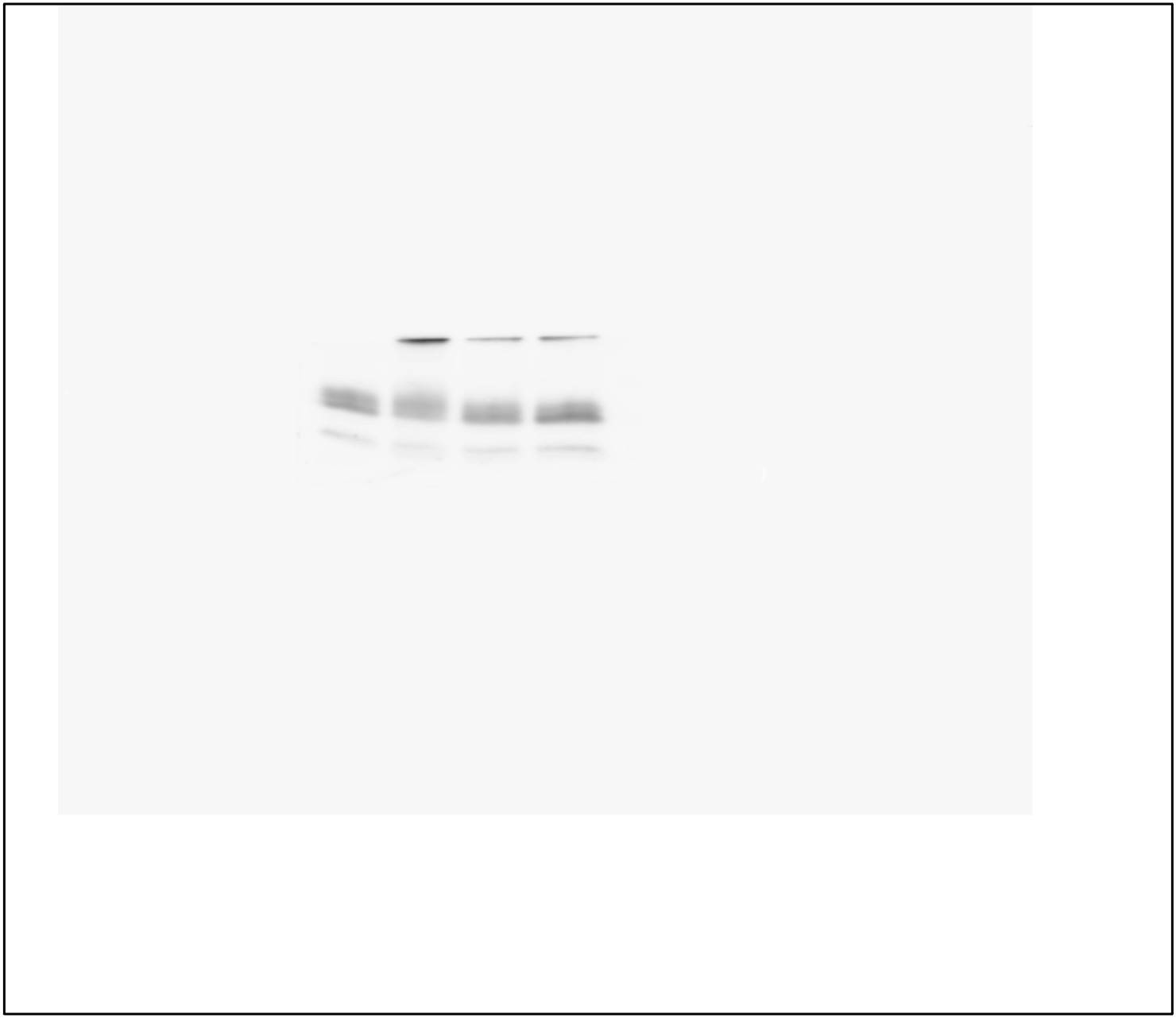

Supplement: Figure 7—figure supplement 2—source data 1. — In the original pictures, the order of lines is the same than in Figure 7—figure supplement 2 and the name of the file indicates the antibody used to develop the nitrocellulose membrane. [file elife-77455-fig7-figsupp2-data1.zip › Supplementary Figure 4 WB/4EB-P1_total.jpg]

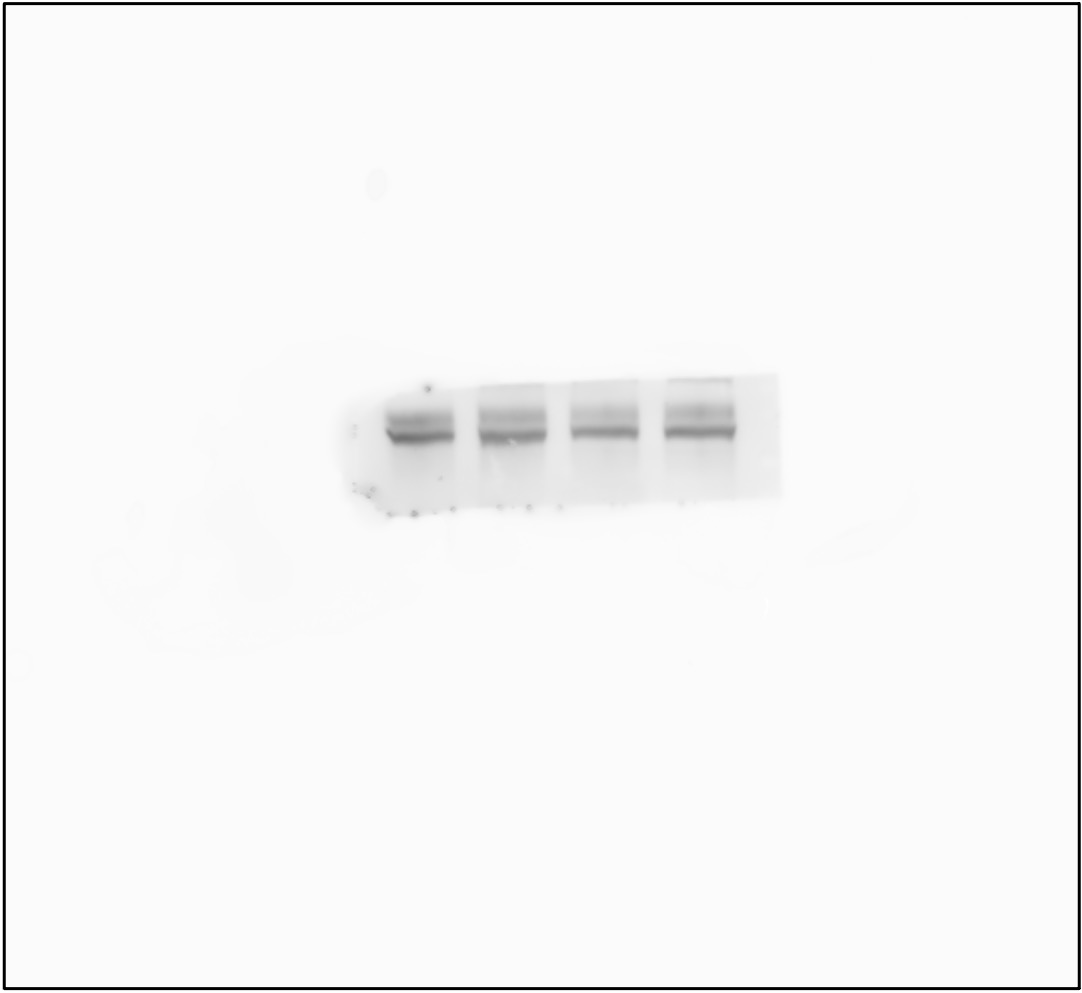

Supplement: Figure 7—figure supplement 2—source data 1. — In the original pictures, the order of lines is the same than in Figure 7—figure supplement 2 and the name of the file indicates the antibody used to develop the nitrocellulose membrane. [file elife-77455-fig7-figsupp2-data1.zip › Supplementary Figure 4 WB/TrkB_total.jpg]

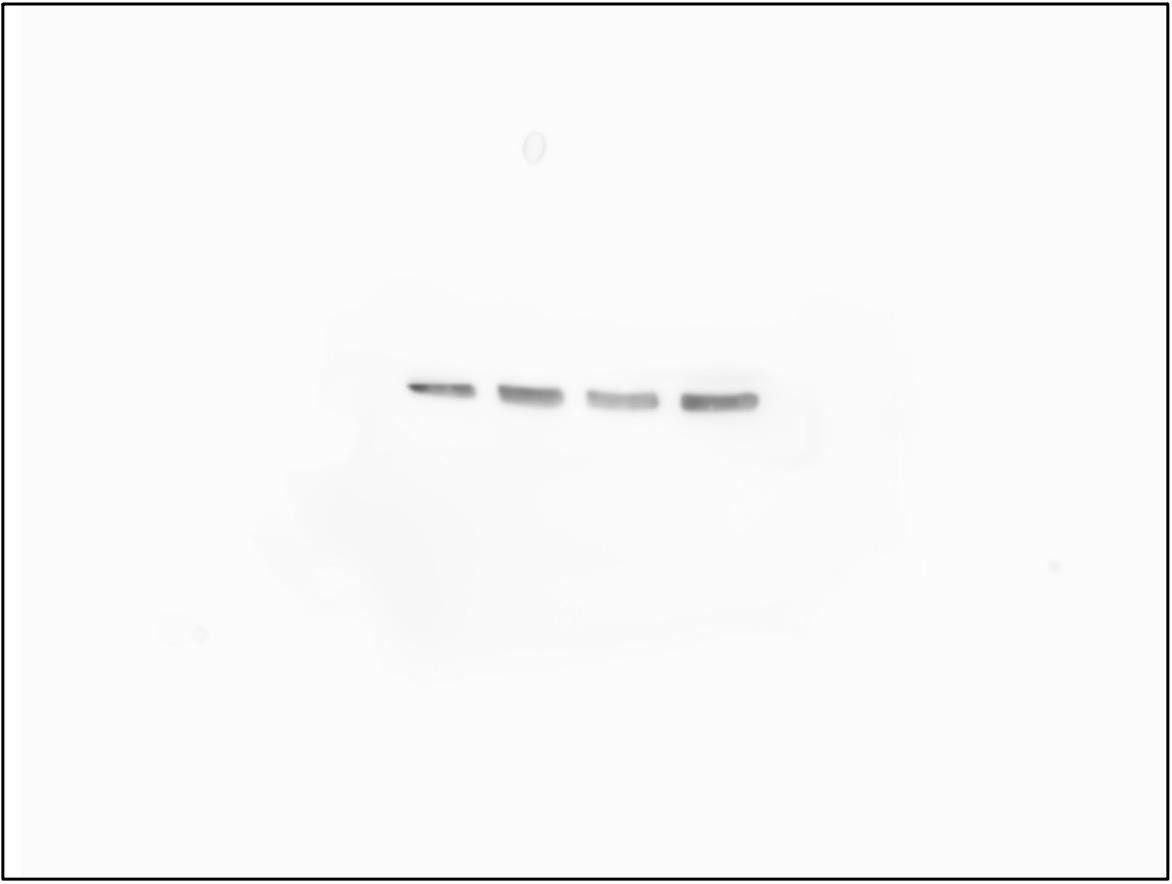

Supplement: Figure 7—figure supplement 2—source data 1. — In the original pictures, the order of lines is the same than in Figure 7—figure supplement 2 and the name of the file indicates the antibody used to develop the nitrocellulose membrane. [file elife-77455-fig7-figsupp2-data1.zip › Supplementary Figure 4 WB/GAPDH.jpg]

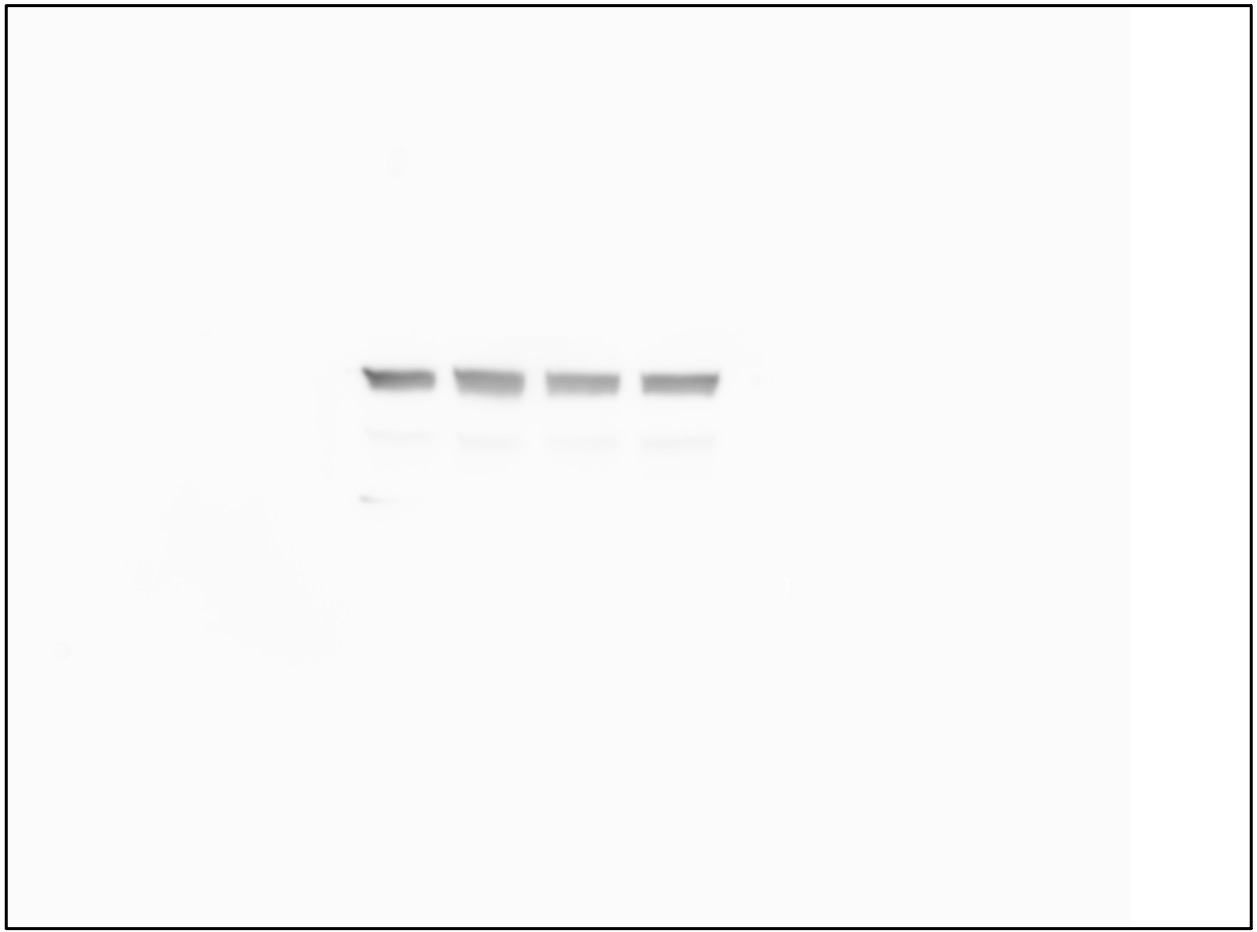

Supplement: Figure 7—figure supplement 2—source data 1. — In the original pictures, the order of lines is the same than in Figure 7—figure supplement 2 and the name of the file indicates the antibody used to develop the nitrocellulose membrane. [file elife-77455-fig7-figsupp2-data1.zip › Supplementary Figure 4 WB/Akt_total.jpg]

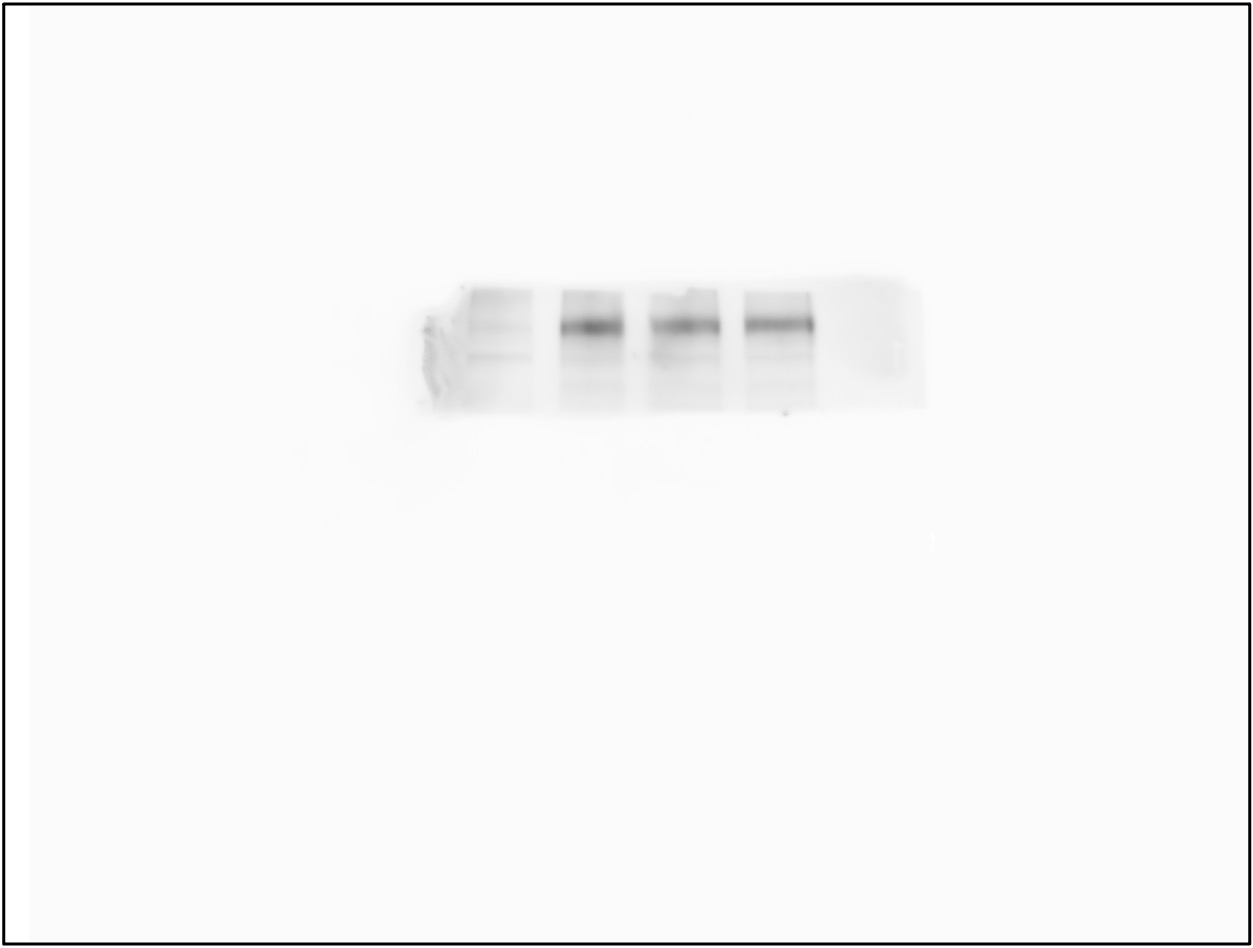

Supplement: Figure 7—figure supplement 2—source data 1. — In the original pictures, the order of lines is the same than in Figure 7—figure supplement 2 and the name of the file indicates the antibody used to develop the nitrocellulose membrane. [file elife-77455-fig7-figsupp2-data1.zip › Supplementary Figure 4 WB/pTrkB515jpg.jpg]
